# Preservation of Ranking Order in the Expression of Human Housekeeping Genes

**DOI:** 10.1371/journal.pone.0029314

**Published:** 2011-12-22

**Authors:** Grace T. W. Shaw, Edward S. C. Shih, Chun-Houh Chen, Ming-Jing Hwang

**Affiliations:** 1 Institute of Biomedical Informatics, National Yang-Ming University, Taipei, Taiwan; 2 Institute of Biomedical Sciences, Academia Sinica, Taipei, Taiwan; 3 Institute of Statistical Science, Academia Sinica, Taipei, Taiwan; 4 Chemical Biology and Molecular Biophysics Program, Taiwan International Graduate Program, Institute of Biological Chemistry, Academia Sinica, Taipei, Taiwan; 5 Institute of Bioinformatics and Structural Biology, National Tsing Hua University, Hsinchu, Taiwan; The Walter and Eliza Hall of Medical Research, Australia

## Abstract

Housekeeping (HK) genes fulfill the basic needs for a cell to survive and function properly. Their ubiquitous expression, originally thought to be constant, can vary from tissue to tissue, but this variation remains largely uncharacterized and it could not be explained by previously identified properties of HK genes such as short gene length and high GC content. By analyzing microarray expression data for human genes, we uncovered a previously unnoted characteristic of HK gene expression, namely that the ranking order of their expression levels tends to be preserved from one tissue to another. Further analysis by tensor product decomposition and pathway stratification identified three main factors of the observed ranking preservation, namely that, compared to those of non-HK (NHK) genes, the expression levels of HK genes show a greater degree of dispersion (less overlap), stableness (a smaller variation in expression between tissues), and correlation of expression. Our results shed light on regulatory mechanisms of HK gene expression that are probably different for different HK genes or pathways, but are consistent and coordinated in different tissues.

## Introduction

Housekeeping (HK) genes are defined as genes that are permanently activated throughout the life cycle of the cell [1]. As they constitute the basic transcriptome for maintaining cellular functions for cell survival, HK genes are also called maintenance genes [2]. In general, genes that participate in essential cellular processes can be considered to have HK functions. These include genes involved in transcription [3], translation [4], [5], energy production and transmission [6], [7], and maintaining cell organization, shape, and motility [8]. HK genes were initially discovered in experiments involving RNA blot hybridization [9] and immunological detection [4], when certain genes were found to be expressed not only constitutively, but also at fairly constant levels under all conditions tested [9]. On the basis of this stable and ubiquitous expression, they have frequently been used as endogenous references for various mRNA quantification experiments [10]–[12]. However, studies have shown that the expression of the HK genes actually fluctuates from tissue to tissue and often from person to person [13], [14]. Furthermore, in disease states, such as liver and breast tumors, HK genes can exhibit very different expression patterns from those observed in normal tissues [15].

While the assumption of constant expression may not be valid, HK genes are useful references so long as their expression patterns are characterized under the same conditions as those in which the experiments are conducted [16], [17]. For example, a stable expression ratio of two HK genes [18], [19] and stable mean value of the expression of several HK genes [11], [20], [21] have been proposed as internal controls in mRNA quantification experiments. However, these propositions are not without flaws, because, for example, the expression ratio of the *RPL32* and *GAPDH* transcripts, two commonly used internal controls in RNase protection assays, is found to fluctuate in mitogen-stimulated mononuclear cells [18]; likewise, a stable mean expression of multiple HK genes in breast tumors is no guarantee that it will be stable in other tissue types [21]. To control for these context-dependent effects, in-advance characterization of HK genes is required, but these characterizations are laborious and time-consuming, so the possibility of finding other common properties of HK genes is of significant interest.

In surveying several large-scale transcriptomics studies in the literature, we noticed that HK genes seemed to follow a similar ranking order in terms of their level of expression in different tissues, even though the actual level might vary from one tissue to another. For example, using data from a study report by Lisowski et al. [22], which investigated stability of gene expression in cattle tissues, we found that, although the expression of six common HK genes, *ACTB*, *GAPDH*, *HPRT1*, *SDHA*, *TBP*, and *YWHAZ*, differed in the kidney, liver, pituitary, and thyroid, the same ranking order of level of expression was seen in all four tissues (Figure S1). In the present study, by performing a statistical analysis of microarray expression data for human genes, we substantiated this observation and showed that an expert-curated set of human HK genes indeed tended to exhibit a preserved tissue-wide expression ranking. Furthermore, we identified the main factors responsible for the preserved ranking and discussed possible underlying mechanisms.

## Results

As detailed in the Methods, based on a manual curation for HK genes [23] and an index for tissue specificity for TS genes [24], we divided the human genes into the two sets of HK and non-HK (NHK) genes, and from the NHK set we selected tissue-specific (TS) genes and assigned the rest as middle-ranged (MR) genes. For the Affymetrix's GSE2361 dataset (Methods and Table S1) used to illustrate the analysis below, this resulted in 388 HK genes and 12,687 NHK genes, and of the NHK genes 734 were TS genes and 11,953 were MR genes. We then computed Kendall's tau [25] for each of the three (HK, MR and TS) gene sets to measure and compare the extent to which the ranking order of gene expression was preserved across tissues. Kendall's tau (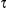) can be computed either on pairs of tissues (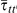) or on pairs of genes (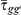); the two are often used interchangeably below, since, in our case, they were essentially identical (see Methods).

### Preservation of expression ranking

**Figure 1:**
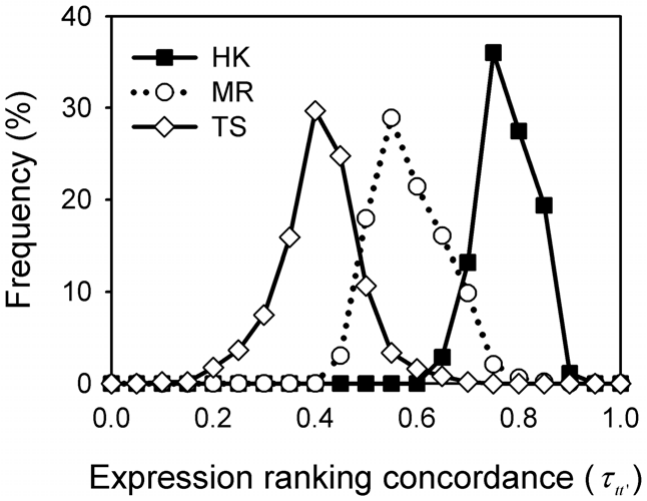
HK genes exhibited a significantly more preserved expression ranking order than MR or TS genes. Frequency is the percentage of tissue pairs with the indicated Kendall's tau (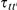). Each distribution is a compilation of 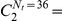
630 Kendall's tau (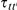; *Equation (3* *)*), where each 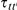 was for a pair of tissues sampled from a 36-tissue pool and the frequency of 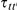 at a particular value was computed on a histogram using a bin width of 0.1. The average value (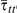) of the 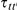 distribution for the HK, MR, and TS gene sets was 0.77, 0.59, and 0.41, respectively, and the standard errors were all small, mostly less than 0.01.

**Figure 2:**
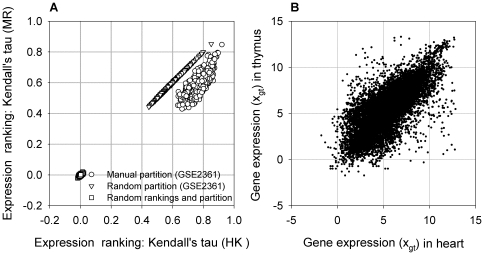
Ranking preservation of HK genes was not a random event. (A) Kendall's tau computed for the manually partitioned HK and MR genes (circles) compared to those generated from two different random distributions: the triangles show the results when the GSE2361 expression data were randomly divided into two sets containing the same number of HK (*N_g_* = 388) and MR (*N_g_* = 11,953) genes, while the squares show the results when expression levels were ignored and rankings were randomly created (see Methods). Each data point is a combination of the two 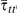 computed for the two gene groups (*N_g_* = 388 and *N_g_* = 11,953) for a particular pair of tissues. (B) The expression of all the genes in any two human tissues (heart and thymus are shown as an example) always has an elliptical shape, resulting in a substantially preserved ranking order with a non-zero 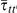 (>0.4) even for randomly grouped genes (triangles in (A)). In contrast, randomly assigned rankings would yield a 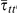 value very close to zero (squares in (A)). Gene expression levels were log2 transformed and were denoted by *x_gt_*.

Figure 1 shows that, as measured by Kendall's tau, the expression ranking order of HK genes in the 36 human tissues of the GSE2361 dataset (Methods and Table S1) was more concordant than those of the MR and TS genes. The 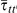 for all pairs of tissues sampled was 0.77 for HK genes, 0.59 for MR genes, and 0.41 for TS genes (Figure 1; Table 1). The non-zero Kendall's tau for the MR genes and, to a lesser extent, for the TS genes suggests some degree of preserved ranking in the expression of the selected genes; in fact, even a randomly sampled set of genes will exhibit a non-zero Kendall's tau (Figure 2A). In general, there was a significant correlation between the expression levels of the same gene in any two tissues, because genes with high expression levels, which are more likely to rank high than low, in one tissue tend to have high expression levels in another tissue (e.g. Figure 2B). Consequently, only when rankings were randomly assigned were truly random (close to zero) Kendall's taus produced (Figure 2A). Compared to MR genes or a randomly selected group of genes, the expression ranking of TS genes varied more between tissues, i.e. producing a smaller 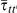, owing to their expression in a specific tissue and no, or little, expression in most other tissues [24].

**Table 1:** Kendall's tau computed for the HK, MR, and TS genes, for four datasets.

Dataset	Data type	Kendall's tau ( 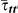 )
	HK	0.77±0.00
GSE2361 [24]	MR	0.59±0.00
	TS	0.41±0.00
	HK	0.69±0.00
GSE1133 [50]	MR	0.47±0.00
	TS	0.34±0.00
	HK	0.79±0.01
GSE803 [51]	MR	0.62±0.01
	TS	0.26±0.00
	HK	0.77±0.00
Human BodyMap 2.0 data [53]	MR	0.59±0.00
	TS	0.42±0.00

Similar results using the same analysis procedures were obtained using two other Affymetrix and one Illumina datasets of human gene expression (Table 1). Thus, in all the three Affymetrix nucleotide microarray datasets and the one Illumina's next-generation RNA sequencing dataset analyzed (Table S1), it was evident that the expression ranking of the selected HK genes across tissues was much more preserved than would be expected if the genes were picked randomly or if the genes were in the NHK set (either the MR or TS set), although the mean value of their Kendall's taus can differ in different expression datasets (Table 1).

### Contributions of the three factors of co-expression, stableness, and dispersion

**Figure 3:**
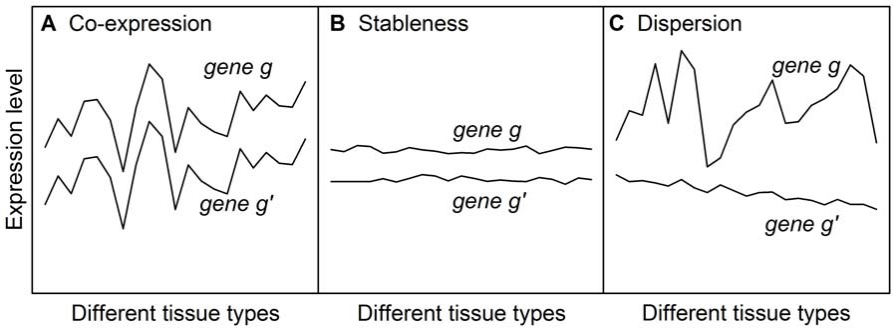
Schematic illustration of the three factors that contribute to preserve gene expression rankings. The three factors represent three different types of expression pattern of a pair of genes in different tissues: (A) Co-expression (*r_gg′_*; *Equation (5* *)*), (B) stableness (*S_gg′_*; *Equation (7* *)*), and (C) dispersion (*D_gg′_*_;_ *Equation (8* *)*).

**Figure 4:**
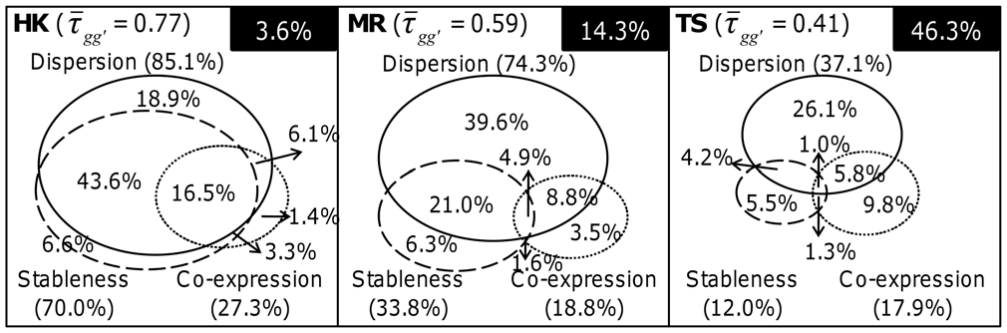
The contributions to the ranking preservation of the HK, MR, and TS genes. The three contributing factors were dispersion (solid line), stableness (dashed line), and co-expression (dotted line). For each factor, its contribution (in parenthesis) was the sum of the individual contributions calculated using tensor product decomposing equations, such as *Equation (11* *)–(13)*. The contribution of factors other than these three is given in the black box in the upper right corner of each panel.

To investigate what produced the observed preservation of expression ranking of the three gene sets, we considered three factors, co-expression, stableness, and dispersion (Figure 3) and carried out a tensor product analysis of a gene pair's Kendall's tau, 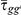, as described in the Methods. Intuitively, when the expression of two genes is highly correlated, the order of their expression levels across tissues will be preserved (Figure 3A). Additionally, when the expression levels of two genes are very stable (Figure 3B) or are highly dispersed (i.e. do not overlap) (Figure 3C), their expression ranking order will also have a high probability of being preserved. As presented in Figure 4, all three factors contributed significantly to the 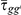 observed for the three gene sets, but their total contribution decreased on going from HK (96.4%) to MR (85.7%) to TS (53.7%); in the case of the TS genes, factors other than the three considered contributed almost as much (46.3%) to their 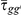 value of 0.41. Of the three factors, dispersion contributed the most, followed by stableness, then co-expression, except in the TS set in which co-expression contributed somewhat more than stableness (17.9% vs. 12%). However, the stableness value showed the largest difference between sets, in that its contribution to the HK set (70%) was more than twice that to the MR set (33.8%) and five times that to the TS set (12%). A joint contribution of two or three factors was particularly marked for the HK genes, suggesting that their expression profiles depended on multiple characteristics to a greater degree than the other two gene sets.

### Stratification by Kyoto Encyclopedia of Genes and Genomes (KEGG) pathways

To investigate whether the preserved ranking order of gene expression resulted from biological regulation, we mapped the genes to KEGG pathways, in which genes performing related functions as categorized by biological pathways are grouped [26].

Of the 198 human pathways annotated in the KEGG, we removed 37 belonging to the disease class and 6 with no more than 2 genes, leaving 155 pathways for analysis. Two hundred and seventy-four of the 388 HK genes were annotated to belong to these pathways, and the vast majority (264, or 96.4%) of these was distributed in 7 pathways; these included 47 genes that were also found in one or more of the remaining 148 pathways. As shown in Table 2, the 7 pathways were enriched in HK genes from the manually curated set, the percentage of HK genes in each of the 7 pathways ranging from 34% to 100%, considerably higher than would be expected from an unbiased sampling of all genes (p values for these percentages were statistically significant, ranging from 0.04 to 0.006; see Table 2). Furthermore, the 7 HK-enriched pathways link molecular biology's central processes, i.e. from gene transcription (Basal Transcription Factors and RNA Polymerase II) to mRNA processing (Spliceosome) to protein translation (Ribosome and Aminoacyl-tRNA Biosynthesis) and degradation (Ubiquitin Mediated Proteolysis and Proteasome). In comparison, of the 12,687 NHK genes (see Methods), only 176 were found in the 7 HK-enriched pathways, while 3,602 were found in the remaining 148 pathways. The enrichment of HK genes in the 7 pathways was not surprising, since KEGG, along with Reactome [27], was used to identify genes with essential cellular functions [23].

**Table 2:** Kendall's tau (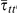) and co-expression (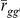) computed for the HK genes in KEGG pathways.

KEGG pathway (number of genes; % in the 388 HK gene set)^a^	Expression ranking^b^ 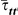 (mean±SE)^d^	Co-expression^c^ 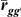 (mean±SE)^d^
Basal Transcription Factors (33; 69.7%)	0.69±0.00	0.22±0.01
RNA Polymerase II (11; 100.0%)	0.70±0.01	0.20±0.01
Spliceosome (113; 46.9%)	0.67±0.00	0.30±0.01
Ribosome (84; 94.1%)	0.75±0.00	0.70±0.00
Aminoacyl-tRNA Biosynthesis (30; 63.3%)	0.66±0.00	0.25±0.01
Ubiquitin-Mediated Proteolysis (117; 34.2%)	0.65±0.00	0.23±0.01
Proteasome (42; 92.9%)	0.59±0.00	0.33±0.01

a Based on the distribution of the percentage of HK genes for each of the 155 KEGG pathways, the p value for a 34% was calculated to be 0.04, and 0.006 for 100%.

b With the exception of the Proteasome pathway, these values are statistically significant (p value<10^−20^ by the paired two-sample Student's t test) compared to the value of 0.58±0.00 obtained for the NHK genes mapped to the remaining 148 KEGG pathways. The p value for the Proteasome pathway was 7.67×10^−4^.

c All these values are statistically significant (p value<10^−20^ by Student's t test) compared to the alternative hypothesis of no correlation.

d SE: standard error.

Table 2 shows that, except for the Proteasome pathway (0.59±0.00), the Kendall's taus (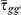) of expression ranking of the HK genes within their respective pathway (0.65±0.00 to 0.75±0.00) were all significantly higher than that (0.58±0.00) of the 3,602 NHK genes in the other 148 pathways, again revealing a statistically significant difference in the preservation of their expression ranking. Furthermore, co-expression correlation (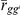) in these HK-enriched pathways was mostly moderate, about 0.2 or 0.3, in accordance with the observation made earlier that co-expression was a smaller contributing factor than either stableness or dispersion to expression ranking (Figure 4). The exception was the Ribosome pathway, which exhibited a high 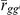 (0.70±0.00) and, as a result, a high 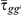 (0.75±0.00). A high 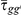 (0.75±0.00) was also obtained for HK genes sampled from different pathways (one from each pathway), which can be attributed to the much larger range of expression levels exhibited between different pathways than within a single pathway (Figure S2).

**Figure 5:**
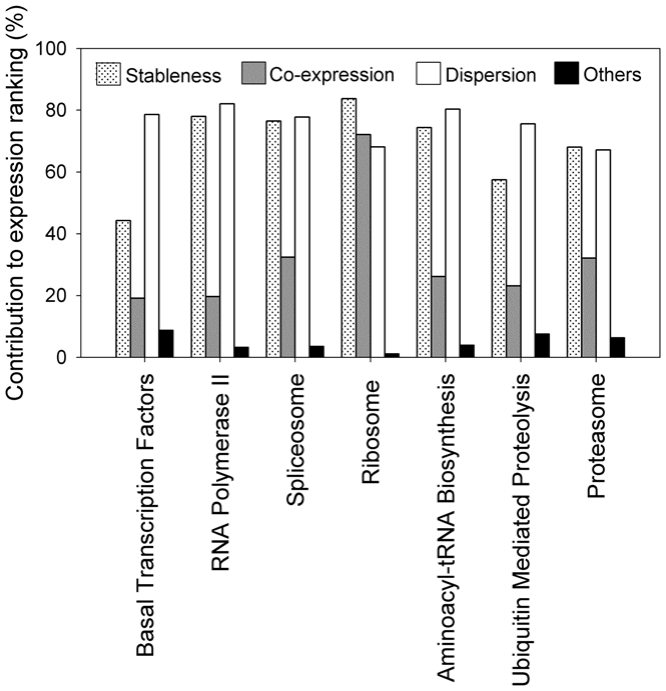
The contributions to the ranking preservation of the HK genes in seven HK-enriched KEGG pathways. Shown is the percentage contribution of the three factors, stableness, co-expression, and dispersion to the expression ranking (Kendall's tau, 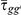) computed for the HK genes found in each of the seven HK-enriched KEGG pathways. The black bars are the contributions of other unknown factors.

Decomposing 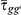 into the three contributing factors for the 7 HK-enriched pathways showed a distribution that was similar to that observed for the 388 HK genes (Figure 4), in that both dispersion and stableness contributed significantly (∼70–80%), while co-expression was a relatively minor factor, with only a ∼20–30% contribution (Figure 5). The high contribution of co-expression in the Ribosome pathway, resulting from a high co-expression correlation, as mentioned above, and the comparatively low contribution of stableness for the Basal Transcription Factors pathway are notable departures that merit further investigation.

**Figure 6:**
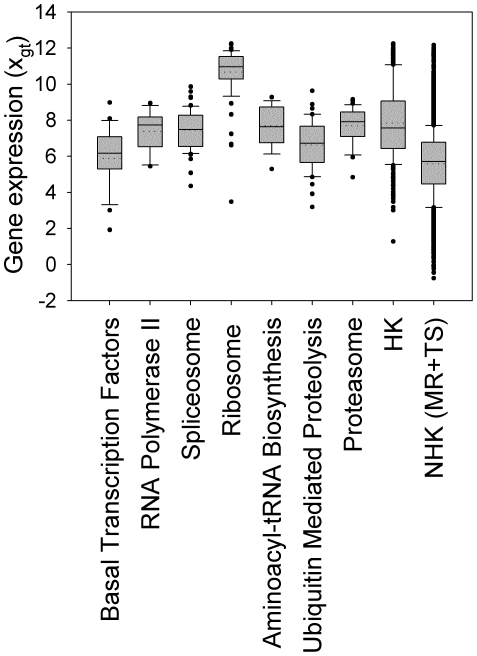
Range of expression levels of the HK genes in HK-enriched KEGG pathways. Shown are boxplots of the tissue-wide expression profiles of the HK genes in each of the 7 HK-enriched KEGG pathways. The results for the whole HK set (388 genes) and for the NHK set (12,687 genes) are presented on the right for comparison. Each box is bounded by the 25^th^ and 75^th^ percentile of the data, with the solid line within the box marking the median and the dotted line the mean, while the two short horizontal bars indicate the 90^th^ and 10^th^ percentile of the data and dots beyond these two bars are outliers. Gene expression levels were log2 transformed and are denoted by *x_gt_*.

Figure 6 shows that the expression levels of HK genes were generally higher than those of NHK genes, an observation also made by others [28]. Stratification of the results into the 7 HK-enriched pathways revealed that, of these HK genes, those in the Ribosome pathway had the highest expression levels and those in the Basal Transcription Factors pathway the lowest. This explains the corresponding highest and lowest contribution of stableness in these two pathways (Figure 5), since high expression levels can withstand expression variation more than low expression levels.

**Figure 7:**
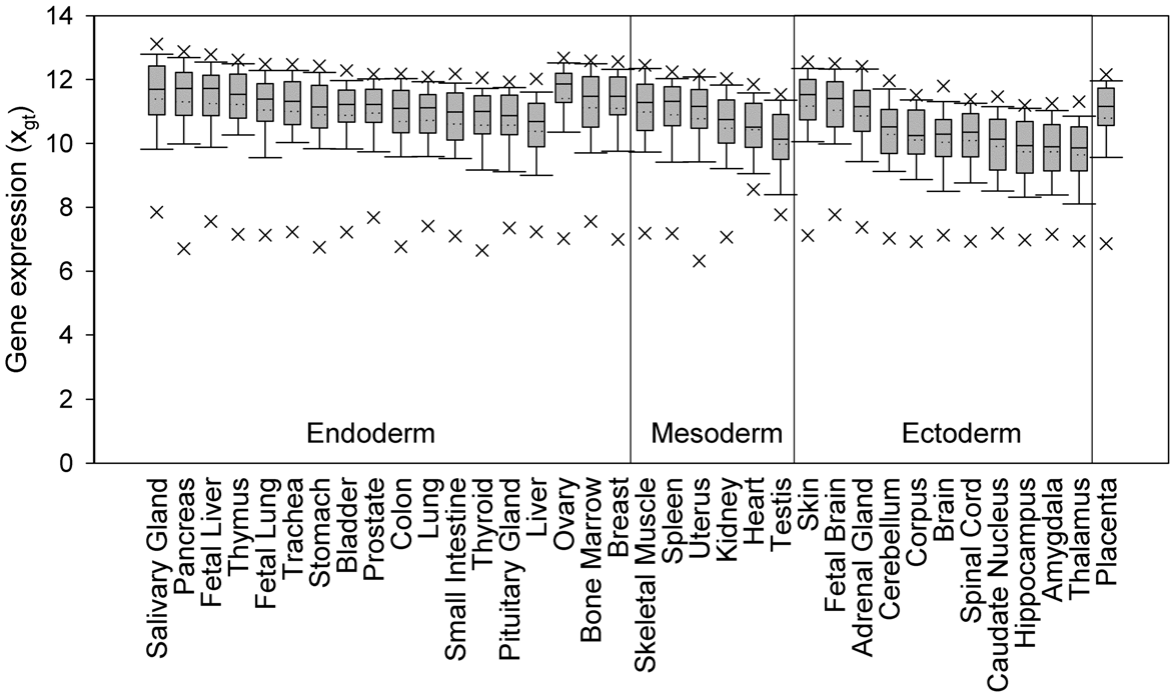
The ribosomal HK genes are expressed at different levels in tissues of different embryonic origins. Shown are boxplots of expression levels on a log2 scale (*x_gt_*) for 79 ribosomal HK genes (94.1% of the 84 ribosomal genes, Table 2) in each human tissue. Each box is bounded by the 25^th^ and 75^th^ percentile of the data, the solid line within the box marking the median. The two short horizontal bars indicate the 90^th^ and 10^th^ percentile of the data, while the crosses mark the 95^th^ and the 5^th^ percentile of the data. The tissues are grouped according to their embryonic origin.

During transcription, the transcription factors of the Basal Transcription Factors pathway mediate the binding of RNA polymerase II to trigger initiation of transcription. Given that initiation is a rapid step in transcription [29], and, as shown in yeast, the transcription of some genes is not dependent on basal transcription factors [30], genes in this pathway may not need to be expressed at high levels. In contrast, the ribosome is necessary for translating all protein-coding genes, and, because translation is a time-consuming process and relies greatly on the cooperation of multiple ribosomal proteins [31], high expression levels of genes in this pathway would be expected. However, interestingly, the 79 HK genes in the Ribosome pathway (Table 2) exhibited different levels of expression in tissues of different embryonic origin (Figure 7), their mean expression levels in tissues with an ectoderm, mesoderm, or endoderm origin being 10.24±0.16, 10.80±0.15, and 10.90±0.08, respectively. Most of the ectoderm-derived tissues constitute the mature nerve system, in which fewer gene products are expressed continuously [32] and transcription of the ribosomal genes need not be as active as in, say, bone marrow, a mesoderm-derived tissue that is a factory generating blood cells, thus requiring continual activation of the ribosomal genes, or the endoderm-derived saliva gland, in which the production of salivary amylase requires high utilization of ribosomes. Consequently, the expression levels of these HK genes in the Ribosome pathway are highly correlated across tissues of different developmental origins, leading to the observed high co-expression correlation (0.70±0.00) (Table 2).

**Figure 8:**
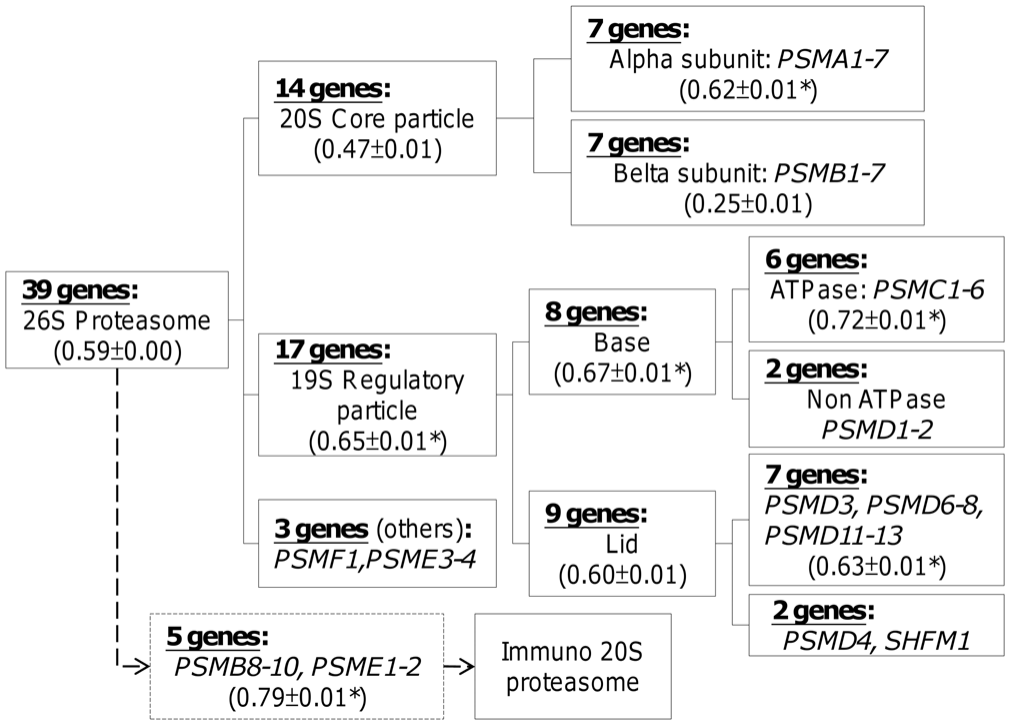
A less coherently regulated subcomplex (β) reduced the overall expression ranking preservation of proteasomal genes. Shown are gene expression rankings (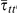) computed for HK genes coding for proteins involved in different subcomplexes of the proteasome. The 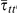 value is shown in parenthesis at the bottom of the box. * indicates that the value is significantly different from 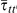 computed from random sampling using the same number of genes.

Finally, we can consider what could be responsible for the low 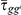 (0.59±0.00) for the Proteasome pathway (Table 2), despite a typical contribution distribution from the three factors (Figure 5) and typical expression levels of HK genes (Figure 6) in this pathway. Further stratification of the Kendall's tau results by the subcomplexes of the proteasome (Figure 8) indicated that the 7 HK genes producing the β subunit of the 20S proteolytic core particle were the culprit. It appears that the expression of not all the proteins in the subcomplexes of proteasome is coherently regulated [33]. For example, while the 7 gene products of the α subunit are needed in equal amounts to form heptamer rings [34], the 7 gene products of the β subunit cannot form rings by themselves [35]. In fact, the 7 β-subunit proteins tend to remain in the monomer state and often exhibit TS expression [36]; as a result, there was a reduced preservation of the ranking order of their expression across tissues, which, in turn, resulted in a decrease in the 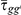 for the Proteasome pathway (Table 2 and Figure 8).

## Discussion

Activation or modulation of regulatory events triggered by different stimuli, such as hormones, transcription factors, or other environmental changes, results in different levels of expression of the same gene in different cells or tissues and, therefore, one would not necessarily expect tissue-wide gene expression profiles to exhibit a preserved ranking order. However, in this study, we showed that the ranking order for the expression levels of the HK genes was significantly more preserved than that of the NHK genes in human tissues (Figure 1; Table 1). This observation was substantiated by using data obtained from different gene expression technologies (oligonucleotide microarray and short-read RNA sequencing; Table 1), as well as an alternative set of HK genes [37] (Figure S3).

Tensor product analysis further revealed that dispersion, which results from minimal overlaps due to, for example, a wide range of expression levels, and stableness, a previously recognized hallmark of HK genes, were two major factors underlying the observed expression ranking preservation, whereas, perhaps unexpectedly, co-expression made a relatively minor contribution (Figures 3 and 4). However, closer examination showed that, in certain pathways, such as the Ribosome pathway (Table 2), or in the highly collaborative expression of the proteins in a subcomplex of a protein complex, such as the α subunit subcomplex (see Figure 8) of the proteasome (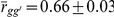, data not shown), co-expression was indeed prominent. Furthermore, to a large extent, the HK genes exhibited preserved tissue-wide expression rankings with contributions from all three factors, especially dispersion and stableness (Figure 4). Together, these results suggest that, compared to NHK genes, HK gene expression is regulated more consistently from tissue to tissue and that different mechanisms may be involved in the regulation of different functional groups of HK genes.

It has been shown in various studies that, compared to NHK genes, HK genes tend to have a shorter coding sequence [37], fewer exons and shorter exons [37], and a higher GC content [38]. However, while these observations are statistically significant as a whole, these properties are poor measures for distinguishing between HK and NHK genes due to the large overlap between the two in terms of these properties (Figure S4). Moreover, it is difficult to reconcile how these static properties of genes could confer the differences in expression level from one tissue to another, let alone the ranking preservation. In this study, we have uncovered a new property of preserved expression ranking in different tissues, in which grouped HK genes and NHK genes show a significant difference. However, some overlaps between HK and MR genes in their expression ranking were also evident (Figure 1), suggesting that, to identify a gene as an HK gene by computational methods, a composite index comprising multiple properties is probably required. Regardless of whether or not the genes considered are HK genes, the use of rank-invariant genes extracted from multiple experiments as normalization references could reduce systemic distortions in microarray data more than conventional treatments [39], [40]. While it may not be practical to use a global rank-invariant set of gene transcripts, which numbers in thousands [40], as references for real-time PCR experiments, a few of highly rank-invariant genes, those within the same pathway (e.g. Ribosome) in particular, may prove to improve the current protocol of such experiments, but validation of this proposition requires experimental investigations. Furthermore, the tensor structure of human HK gene expressions uncovered in this work (Figure 4) can be useful features for machine learning techniques to develop a classification scheme for discovering novel HK genes (work in progress).

The differences in HK gene expression in different tissues may arise for a number of reasons. One is that the specific function of tissues may dictate the level at which a HK gene needs to be expressed. For example, and as shown in Figure 7, ribosomal genes are expressed at a higher level in bone marrow cells, presumably due to the demand to constitutively refill new blood cells, than in mature nerve cells, which undergo no, or little, regeneration [32]. A second is that many HK genes are among the ∼90% of human genes that are processed by alternative splicing [41], resulting not only in the HK transcript, but also in TS transcripts with TS functions. Consequently, it is possible that, in tissues in which TS transcripts of the HK gene are needed, there exists a distinct regulatory mechanism to balance the expression of the two types of transcripts. A third reason is that mRNA decay rates can vary significantly in different tissues [42].

Nevertheless, our analysis indicated that, despite fluctuations, the HK genes exhibited a high stableness in their expression profiles (Figure 4). This is, in part, due to the relatively high expression levels of HK genes (Figure 6), which, for the same stableness value, can have a larger variation. Intricate regulatory processes may also be at work. As support for this, cell-to-cell noise in the expression of genes encoding protein complexes or with essential biological roles has been shown to be minimized [43]. In addition, many HK genes play a role in gene regulation, with some regulating their own expression. Examples include (i) over 30 proteins in the spliceosome complex have known, or putative, roles in various steps in gene expression [44], (ii) RNA polymerase II transcribes miRNAs to silence gene expression [45] and, through a Rpb4/7 heterodimer in the cytoplasm of yeast, is involved in the mRNA decay pathway [46], (iii) the expression of the constitutive (α) form of the glucocorticoid receptor (GR) is inhibited or enhanced, respectively, by the expression of the alternative GR-β or GR-P transcript by the activation of alternative promoters [47], and (iv) the alternative transcripts of some ribosomal protein genes, e.g. *RPL3* and *RPL12*, are natural targets for nonsense-mediated mRNA decay [48], which can negatively autoregulate their overproduction.

In this study, we have uncovered a novel property of human HK genes, i.e. their significantly preserved expression ranking order in different tissues. Unlike some of the previously identified properties of HK genes, which are static DNA composition and structure of a single gene [21], [37], [49] (Figure S4), the property of expression ranking discovered here is a collective property of a group of genes preserved in different tissues and thus reflects a consequence of tight regulations. Although many HK genes, in addition to their HK functions, have been shown to play a role in various aspects of gene regulation, the exact molecular mechanisms involved in coordinating the apparently tight-regulated expression of HK genes require further studies.

## Materials and Methods

### Human gene expression datasets

We used publicly available Affymetrix microarray data. The oligonucleotide microarray series matrix files derived from Su et al. [50] (GSE1133), Yanai et al. [51] (GSE803), and Ge et al. [24] (GSE2361) were downloaded from GEO (Gene Expression Omnibus) depositories [52]. Unless otherwise noted, GSE2361 was used as the example to describe the procedures of the analysis. The other two datasets, GSE803 and GSE1133, were analyzed by the same procedures to rule out any potential bias of using a single dataset. For an examination on the effect of using data from a different gene expression technology platform, a Human BodyMap 2.0 RNAseq dataset from Illumina recently added to Ensemble release 62 [53] was also analyzed. These four sets of human gene expression data are summarized in Table S1.

### Data processing

Each Affimatrix dataset was processed following the default preprocessing and normalization setups as described in the original articles [24], [50], [51]. Transcripts were then mapped to genes. For example, the expression profiles of the 22,283 transcripts of the GSE2361 and GSE1133 datasets were reduced to a set of 13,075 non-redundant genes by mapping using the Entrez Gene ID [54]. In the case of GSE803, 63,174 transcripts were mapped to 18,592 genes. During the mapping, the expression levels of probe sets, i.e. transcripts, with the same Entrez Gene ID were averaged to represent the expression level of the gene.

The Illumina's sequences were mapped to Refseq genes (i.e. Entrez Gene ID [54]) using RNASEQR, a new short-read RNA sequence mapping tool, and expression levels in RPKM (reads per kilo per million) were calculated (Leslie Chen, personal communication).

### Division of the genes into three groups

In this study, a gene was designated as either HK or NHK, and the NHK genes were subdivided into MR or TS, using the following partitioning procedures. First, the 13,075 non-redundant genes of GSE2361 were divided into two groups, a HK set and a NHK set, based on a study [23] in which 408 genes were found to have well-documented HK functions as annotated in Reactome [27] and the KEGG [26]. Of these 408 manually annotated HK genes, 388 were found in GSE2361 and were placed in the HK set, while the remaining 12,687 genes were placed in the NHK set. The 734 genes of the NHK set that satisfied four criteria for TS genes [24] were then designated as TS genes and the remaining 11,953 NHK genes were placed in the MR set. The same procedure was followed for the other expression datasets analyzed.

### Preservation of gene expression ranking as measured by Kendall's tau

Kendall's tau [25], defined below, was used to measure the extent of concordance in the ranking order of expression levels for a group of genes in different tissues.

Let *x_gt_* denote the log2 transformed expression level of gene *g* in tissue *t*, where *g = 1,2,…,G* and *t = 1,2,…,T* for a total of *G* genes in a total of *T* tissues. *x_gt_* thus represents an element in the *G*×*T* matrix. For any two genes *g* and *g′* in tissues *t* and *t′*, whether their expression levels are concordant or discordant can be computed by:
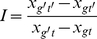
(1)


The pair of genes *g* and *g′* show concordance in the ranking order of their expression levels in tissues *t* and *t′* if *I*>0 and are a discordance pair if *I*<0. Their ranking orders are identical in tissue *t′* if *I* = 0 or in tissue *t* if *I*→∞. Collecting the number of all the *I*>0, *I*<0, *I* = 0, and *I*→∞ cases, denoted by *N_I>0_*, *N_I<0_*, *N_I = 0_*, and *N_I→∞_*, respectively, Kendall's tau (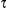) can be computed as follows:
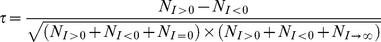
(2)


For a group of *N_g_* genes in *N_t_* tissues (2*≦N_g_≦G* and 2*≦N_t_≦T*), 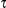 can be computed by summing *Equation (2* *)* either over tissue pairs (*Equation (3* *)*) or over gene pairs (*Equation (4* *)*) and taking the average. Note that, for two tissues only, 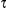 is bounded by −1 and 1, with 1 and −1 representing a perfectly preserved ranking order in the same (1) or opposite (−1) direction, and 0 a completely random ordering. However, as the number of tissues increases, mathematically, the lower boundary would increase from −1 to −0.0286 for the case of *N_t_* = 36.
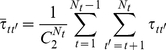
(3)

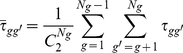
(4)


Strictly speaking, 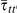 and 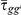 are not identical unless there are no identical rankings (i.e. both *N_I = 0_* and *N_I→∞_* are zero). However, in the expression datasets analyzed, there was no gene pair with an identical ranking in the HK set, and very few (less than 0.5% of gene pairs) in the MR and TS sets, making 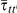 identical, or almost identical, to 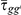.

We computed both 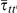 and 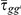 for the three gene sets (HK, MR, and TS), using 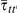 to test the hypothesis that HK genes, as compared to MR or TS genes, tend to have a preserved ranking order of expression levels in different tissues, and 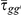, which is easier to decompose, to identify the factors that contribute to the ranking preservation (see the next section).

As a control for comparison, we generated two randomly distributed gene expression rankings. In the first, we randomly sampled *N_g_* genes from the expression dataset, e.g. GSE2361, and calculated 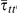 (*Equation (3* *)*) for this group of genes (*N_g_* = 388 when the comparison was made with HK genes, *N_g_* = 11,953 with MR genes and *N_g_* = 734 with TS genes). This was repeated 100,000 times, and the 100,000 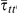 generated were averaged and compared to the 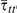 computed from the HK set (or the MR and the TS set). In the second, the sampling procedure was identical, but, instead of using the expression data to produce ranking orders, we created a matrix of randomly assigned rankings with the same dimension of the expression dataset (e.g. 13,075 genes ×36 tissues for the GSE2361 set), in which each column of the matrix, i.e. tissue, contained a randomly assigned string of integers ranging from 1 to the total number of genes (13,075 for the GSE2361 set), and 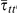 was calculated from this integer matrix.

### Factors involved in ranking preservation

We analyzed three factors that might play a role in preserving/disturbing the ranking order of gene expression in different tissues: these were co-expression, stableness, and dispersion and are schematically illustrated in Figure 3. Below, we devised three measures, all made to range from 0 to 1, for the three factors, respectively.

#### Co-expression (r_gg′_)

As shown in *Equation (5* *)*, the absolute value of the Pearson correlation (*r_gg′_*) [55], [56] was used, since both positive and negative correlations contribute to the ranking order of gene expression. More correlated expressions, i.e. a larger *r_gg′_*, will generally have a more preserved ranking order.
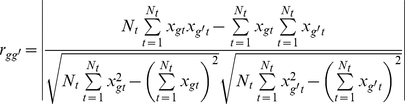
(5)


#### Stableness (S_gg′_)

Let *CV_g_* and *CV_g′_* be the two coefficients of variation for the expression of genes *g* and *g′*, respectively, and *PCV_g_* (or *PCV_g′_*) be the percentage of genes for which *CV* does not exceed *CV_g_* (or *CV_g′_*), as defined by *Equation (6* *)*, then stableness is defined by *Equation (7* *)*.
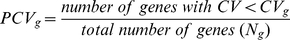
(6)

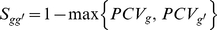
(7)


*Equation (7* *)* dictates that a pair of genes showing very stable expression in different tissues (i.e. a very small *CV*) will have a stableness measure, *S_gg′_*, close to 1.

#### Dispersion (D_gg′_)

Let the highest and lowest expression levels for genes *g* and *g′* in *T* tissues be *Max_g_* = max (*x_g1_*, *x_g2_*, …, *x_gt_*, …, *x_gT_*), *Min_g_* = min (*x_g1_*, *x_g2_*, …, *x_gt_*, …, *x_gT_*), *Max_g′_* = max (*x_g′1_*, *x_g′2_*, …, *x_g′t_*, …, *x_g′T_*) and *Min_g′_* = min (*x_g′1_*, *x_g′2_*, …, *x_g′t_*, …, *x_g′T_*). Supposing that *Max_g_* is greater than *Max_g′_*, the dispersion measure is defined as:
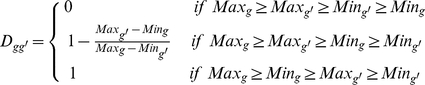
(8)


In general, a larger *D_gg′_*, meaning less overlap between the expression profiles of the two genes, will tend to yield a similar relative ranking order, with *D_gg′_* = 1 providing a guarantee of a perfectly preserved ranking order.

### Venn diagram decompositions

To dissect the intertwined relationships between the aforementioned three factors and the observed ranking of gene expression, we employed the concept of tensor products, which has increasingly been applied to diverse research fields in which multiway data analysis is needed [57], [58].

**Figure 9:**
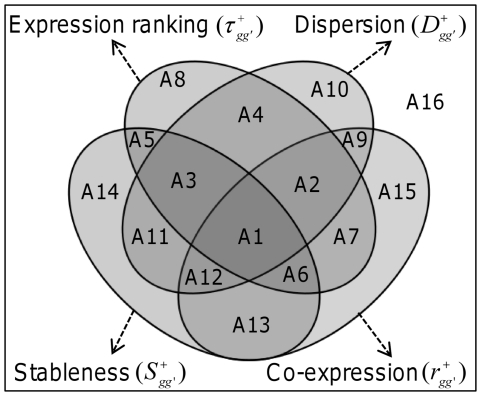
The relationships of the contributing components of gene expression rankings. The four-circle Venn diagram is used to illustrate the intertwined relationships between gene expression ranking (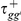) and the three contributing factors of co-expression (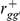, *Equation (5* *)*), stableness (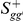, *Equation (7* *)*), and dispersion (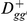, *Equation (8* *)*). The rectangular box represents the universal set, its 16 components can be deduced from tensor computations (*Equation (9* *)*), e.g., A8 = 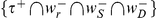, A10 = 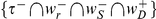, and A16 = 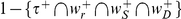. The equations for computing these components are described in the Methods (*Equation (11* *–* *13* *)*). Note that each of the four elements (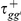,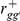,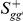, and 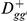) is a composite of eight components.

As illustrated by the Venn diagram [59] shown in Figure 9, the universal set (represented by unity) consists of a composite of 16 components (A1–A16), which relate 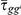 and the three factors. The 16 components can be computed by operations of tensor products (*Equation (9* *)*), where 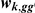 (*Equation (10* *)*) is either 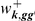 or 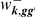, representing the presence or absence of a contributing factor *k*, and *I_8×1_* is an identity vector which, when used in the inner product operation (the big black dot), leads to the separation of the 16 components. Note that, since the theoretical lower boundary of 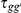 was very close to zero for the data analyzed in our study, as mentioned above, we can assume 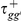
∼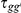 (*Equation (4* *)*) and define 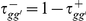 to represent the absence of expression ranking for components A9–A16. Below, for clarity, we have often omitted the symbol (overhead bar) used for the average of all gene or tissue pairs.
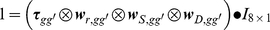
(9)

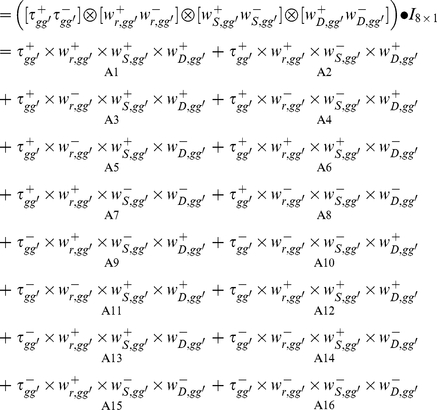


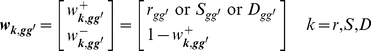
(10)


Thus, for example, component A1 consists of the concurrent contributions of co-expression (*r_gg′_*), stableness (*S_gg′_*), and dispersion (*D_gg′_*) to 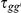, while A2, with the absence of contribution from stableness, consists only of contribution from co-expression and dispersion to 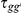.

For a given set of genes, such as those in the designated HK set (or MR and TS set), the contribution of a particular factor, say 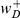, to the observed ranking (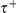) of the expressions of these genes can be computed from *Equation (11* *)*.
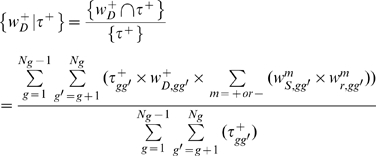
(11)


Likewise, the joint contribution of any two factors, say 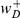 and 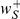, and of the three factors, to 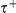 are:
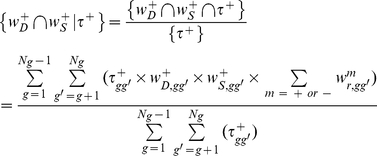
(12)

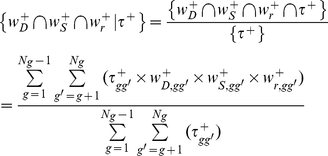
(13)


## Supporting Information

Figure S1 **Expression ranking preservation of HK genes in cattle tissues.** The thresholds (C_t_) for the cycle numbers of real-time PCR experiments for six HK genes in cattle tissues showed rank preservation. In general, C_t_ is negatively correlated with gene expression level. Data from Lisowski et al. [22].
(PDF)

Figure S2
**HK genes selected from different pathways span a wider range of expression levels.** The expression ranges of HK genes in each of the 7 HK-enriched pathways for two tissues are shown by mean value (small solid circles) and standard errors (error bars) of their expression levels in log2 scale. The Kendall's tau, 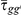 for HK genes selected from different pathways was computed to be 0.75±0.00 for 100 runs of random sampling of 7 HK genes, each from one of the 7 HK-enriched pathways.(PDF)

Figure S3
**Kendall's tau (** 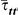 ) **of expression rankings computed for various groupings of genes.** Kendall's tau for genes unique to the HK gene set curated by Zhu et al. [23] was higher than that for genes unique to an alternative set [37] (0.73 vs. 0.66), but both were considerably higher than that for a randomly selected set of MR genes (0.58) and Kendall's tau was the highest (0.79) for genes common to both HK sets. Standard errors for these Kendall's taus were all small, mostly less than 0.01.
(PDF)

Figure S4
**Kendall's tau (** 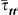 ) **for expression rankings as a function of four gene properties.** The four properties examined are coding sequence (CDS) length (A), number of exons (B), average exon length (C), and GC content (D). The plots for the HK, MR, and TS sets are labeled. Pearson correlations (r_HK_, r_MR_, and r_TS_) for each property are given at the bottom right of each panel. The three horizontal dashed lines represent the average Kendall's tau computed for 100 genes chosen randomly from each of the three gene sets; from top to bottom, these correspond to the HK, MR, and TS sets. Note that, although the correlations for HK genes are high, a threshold cannot be established for any of the four properties to separate HK genes and NHK genes.
(PDF)

Table S1 **The three Affymetrix oligonucleotide microarray datasets and a Human BodyMap 2.0 RNAseq dataset.**
(PDF)
