# Supplementary material for: Preservation of Ranking Order in the Expression of Human Housekeeping Genes
Source: PLoS One. 2011 Dec 22;6(12):e29314. doi: 10.1371/journal.pone.0029314 (PMC3245260; doi:10.1371/journal.pone.0029314)
Supplement: Figure S1 — Expression ranking preservation of HK genes in cattle tissues. The thresholds (Ct) for the cycle numbers of real-time PCR experiments for six HK genes in cattle tissues showed rank preservation. In general, Ct is negatively correlated with gene expression level. Data from Lisowski et al. [22]. (PDF) [file pone.0029314.s001.pdf]

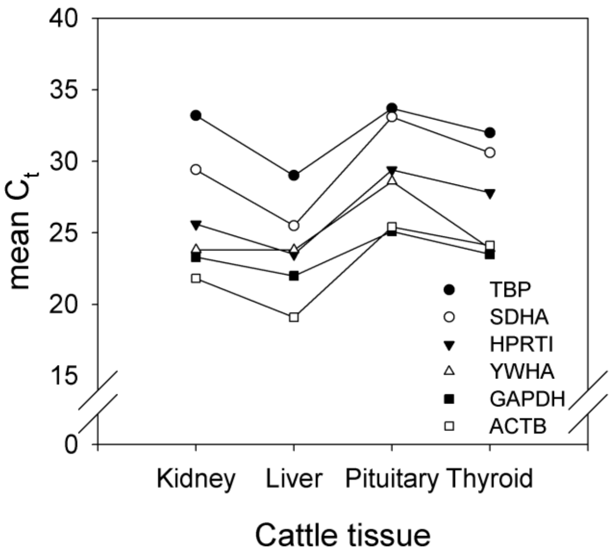

Figure S1. Expression ranking preservation of HK genes in cattle tissues. The thresholds (C_t) for the cycle numbers of real-time PCR experiments for six HK genes in cattle tissues showed rank preservation. In general, C_t is negatively correlated with gene expression level. Data from Lisowski et al. [22].
