# Supplementary material for: Preservation of Ranking Order in the Expression of Human Housekeeping Genes
Source: PLoS One. 2011 Dec 22;6(12):e29314. doi: 10.1371/journal.pone.0029314 (PMC3245260; doi:10.1371/journal.pone.0029314)
Supplement: Figure S2 — HK genes selected from different pathways span a wider range of expression levels. The expression ranges of HK genes in each of the 7 HK-enriched pathways for two tissues are shown by mean value (small solid circles) and standard errors (error bars) of their expression levels in log2 scale. The Kendall's tau, for HK genes selected from different pathways was computed to be 0.75±0.00 for 100 runs of random sampling of 7 HK genes, each from one of the 7 HK-enriched pathways. (PDF) [file pone.0029314.s002.pdf]

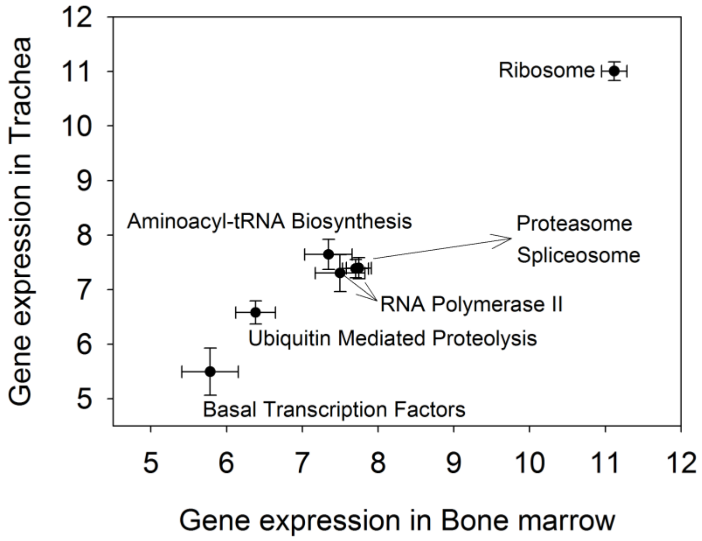

Figure S2. HK genes selected from different pathways span a wider range of expression levels. The expression ranges of HK genes in each of the 7 HK-enriched pathways for two tissues are shown by mean value (small solid circles) and standard errors (error bars) of their expression levels in log2 scale. The Kendall's tau, $\bar{\tau}_{gg'}$ for HK genes selected from different pathways was computed to be 0.75±0.00 for 100 runs of random sampling of 7 HK genes, each from one of the 7 HK-enriched pathways.
