# Supplementary material for: Preservation of Ranking Order in the Expression of Human Housekeeping Genes
Source: PLoS One. 2011 Dec 22;6(12):e29314. doi: 10.1371/journal.pone.0029314 (PMC3245260; doi:10.1371/journal.pone.0029314)
Supplement: Figure S3 — Kendall's tau ( ) of expression rankings computed for various groupings of genes. Kendall's tau for genes unique to the HK gene set curated by Zhu et al. [23] was higher than that for genes unique to an alternative set [37] (0.73 vs. 0.66), but both were considerably higher than that for a randomly selected set of MR genes (0.58) and Kendall's tau was the highest (0.79) for genes common to both HK sets. Standard errors for these Kendall's taus were all small, mostly less than 0.01. (PDF) [file pone.0029314.s003.pdf]

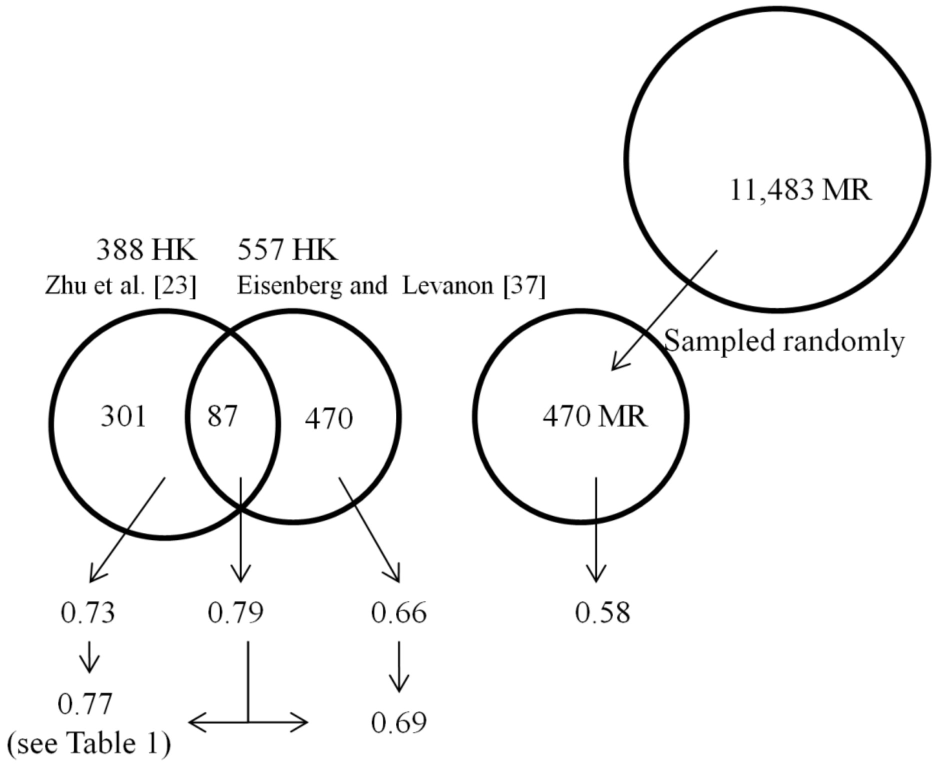

Figure S3. Kendall's tau ($\bar{\tau}_{tt'}$) of expression rankings computed for various groupings of genes. Kendall's tau for genes unique to the HK gene set curated by Zhu et al. [23] was higher than that for genes unique to an alternative set [37] (0.73 vs. 0.66), but both were considerably higher than that for a randomly selected set of MR genes (0.58) and Kendall's tau was the highest (0.79) for genes common to both HK sets. Standard errors for these Kendall's taus were all small, mostly less than 0.01.
