# Supplementary material for: Preservation of Ranking Order in the Expression of Human Housekeeping Genes
Source: PLoS One. 2011 Dec 22;6(12):e29314. doi: 10.1371/journal.pone.0029314 (PMC3245260; doi:10.1371/journal.pone.0029314)
Supplement: Figure S4 — Kendall's tau ( ) for expression rankings as a function of four gene properties. The four properties examined are coding sequence (CDS) length (A), number of exons (B), average exon length (C), and GC content (D). The plots for the HK, MR, and TS sets are labeled. Pearson correlations (rHK, rMR, and rTS) for each property are given at the bottom right of each panel. The three horizontal dashed lines represent the average Kendall's tau computed for 100 genes chosen randomly from each of the three gene sets; from top to bottom, these correspond to the HK, MR, and TS sets. Note that, although the correlations for HK genes are high, a threshold cannot be established for any of the four properties to separate HK genes and NHK genes. (PDF) [file pone.0029314.s004.pdf]

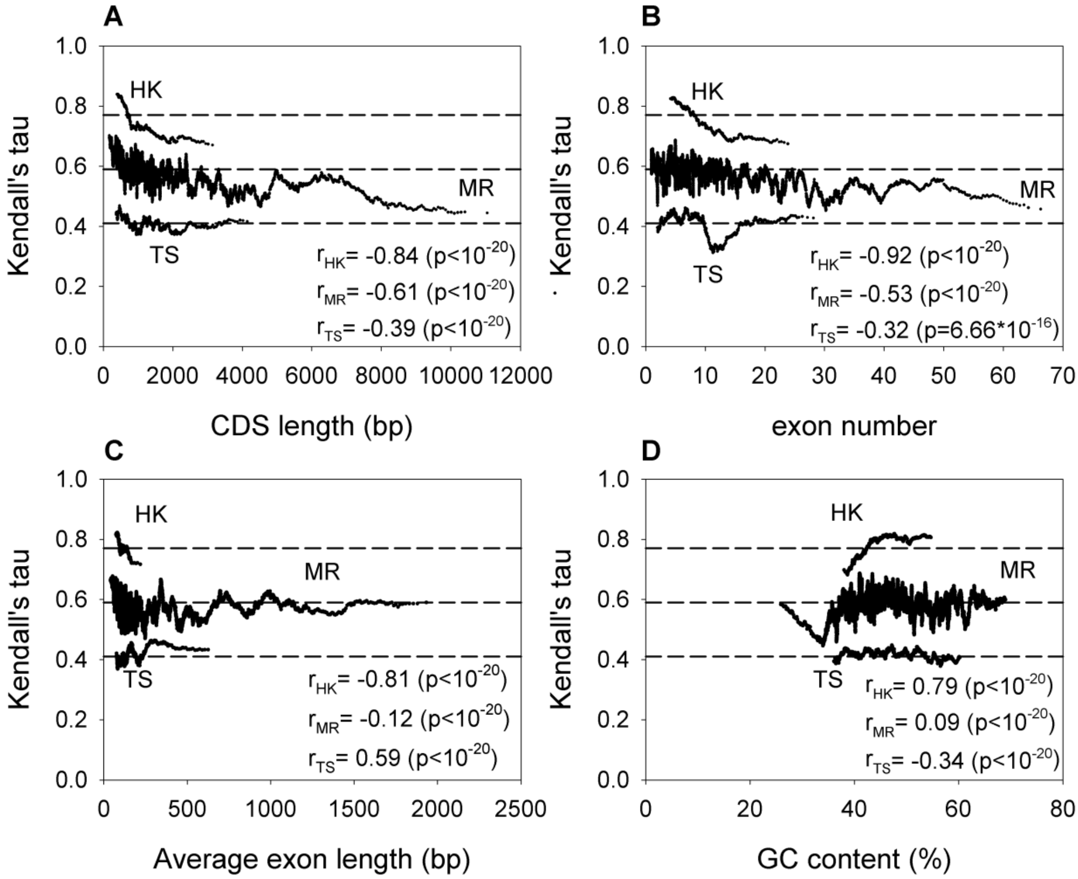

Figure S4. **Kendall's tau ($\bar{\tau}_{tt'}$) for expression rankings as a function of four gene properties.** The four properties examined are coding sequence (CDS) length (A), number of exons (B), average exon length (C), and GC content (D). The plots for the HK, MR, and TS sets are labeled. Pearson correlations (r_{HK}, r_{MR}, and r_{TS}) for each property are given at the bottom right of each panel. The three horizontal dashed lines represent the average Kendall's tau computed for 100 genes chosen randomly from each of the three gene sets; from top to bottom, these correspond to the HK, MR, and TS sets. Note that, although the correlations for HK genes are high, a threshold cannot be established for any of the four properties to separate HK genes and NHK genes.
