# Supplementary material for: Preservation of Ranking Order in the Expression of Human Housekeeping Genes
Source: PLoS One. 2011 Dec 22;6(12):e29314. doi: 10.1371/journal.pone.0029314 (PMC3245260; doi:10.1371/journal.pone.0029314)
Supplement: Table S1 — The three Affymetrix oligonucleotide microarray datasets and a Human BodyMap 2.0 RNAseq dataset. (PDF) [file pone.0029314.s005.pdf]

Table S1. The three Affymetrix oligonucleotide microarray datasets and a Human BodyMap 2.0 RNAseq dataset.

Dataset	Platforms	Transcripts	Tissues
GSE2361 [24]	Affymetrix GeneChip® Human Genome U133 Array Set HG-U133A	22,283	36 normal human tissues
GSE1133 [50]	Affymetrix GeneChip® Human Genome U133 Array Set HG-U133A	22,283	79 normal human tissues
GSE803 [51]	Affymetrix GeneChip® Human Genome U95 Set HG-U95A-E	63,174	12 normal human tissues
Human BodyMap 2.0 RNAseq track in Ensembl release v62 [53]	Illumina HiSeq 2000	34,475	16 normal human tissues
